# Radiographic and clinical outcome of lateral lumbar interbody fusion for extreme lumbar spinal stenosis of Schizas grade D: a retrospective study

**DOI:** 10.1186/s12891-020-03282-6

**Published:** 2020-04-20

**Authors:** Jun Li, Hao Li, Ning Zhang, Zhi-wei Wang, Teng-fei Zhao, Lin-wei Chen, Gang Chen, Qi-xin Chen, Fang-cai Li

**Affiliations:** Department of Orthopedics, the Second Affiliated Hospital, School of Medicine, Zhejiang University, No.88 Jiefang Road, Hangzhou, 310009 China

**Keywords:** LLIF ;lumbar ;spinal stenosis ;indirect decompression, Radiographical outcomes, Clinical outcomes

## Abstract

**Background:**

Extreme lumbar spinal stenosis was thought to be a relative contraindication for lateral lumbar interbody fusion (LLIF) and was excluded in most studies. This is a retrospective study to analyze the radiographic and clinical outcome of LLIF for extreme lumbar spinal stenosis of Schizas grade D.

**Methods:**

For radiographic analysis, we included 181 segments from 110 patients who underwent LLIF between June 2017 and December 2018. Lumbar spinal stenosis was graded according to Schizas’ classification. Anterior and posterior disc heights, disc angle, foramen height, spinal canal diameter and central canal area were measured on CT and MRI. For clinical analysis, 18 patients with at least one segment of grade D were included. Visual analogue scale (VAS) and Oswestry disability index (ODI) scores were used to evaluate clinical outcome. Continuous variables were compared using Student’s t-test, with *P-*values < 0.05 considered to indicate statistically significant differences.

**Results:**

Among the 181 segments included for radiological evaluation, there were 23 grade A segments, 37 grade B segments, 103 grade C segments and 18 grade D segments. Postoperatively, the average change of midsagittal canal diameter of grade D was significantly greater than that of grade A, and not significantly different compared to grades B and C. As to the average change of disc height, bilateral foraminal height, disc angle and central canal area (CCA), grade D was not significantly different from the others. The average postoperative CCA of grade D was significantly smaller than the average preoperative CCA of grade C. Eighteen patients with grade D stenosis were followed up for an average of 19.61 ± 6.32 months. Clinical evaluation revealed an average improvement in the ODI and VAS scores for back and leg pain by 20.77%, 3.67 and 4.15 points, respectively. Sixteen of 18 segments with grade D underwent posterior decompression.

**Conclusion:**

The radiographic decompression effect of LLIF for Schizas grade D segments was comparable with that of other grades. Posterior decompression was necessary for LLIF to achieve a satisfactory clinical outcome for extreme lumbar spinal stenosis of Schizas grade D.

## Background

As a minimally-invasive technique, lateral lumbar interbody fusion (LLIF) has become the first choice of many spine surgeons in recent years. LLIF is capable of restoring foraminal and intervertebral height, thecal sac area, and alignment, with less trauma and lower approach-related morbidity compared with traditional open decompression techniques [1, 2], making it especially suitable for elderly patients, patients with multi-level lumbar spine diseases and patients who cannot tolerate large operations.

**Fig. 1:**
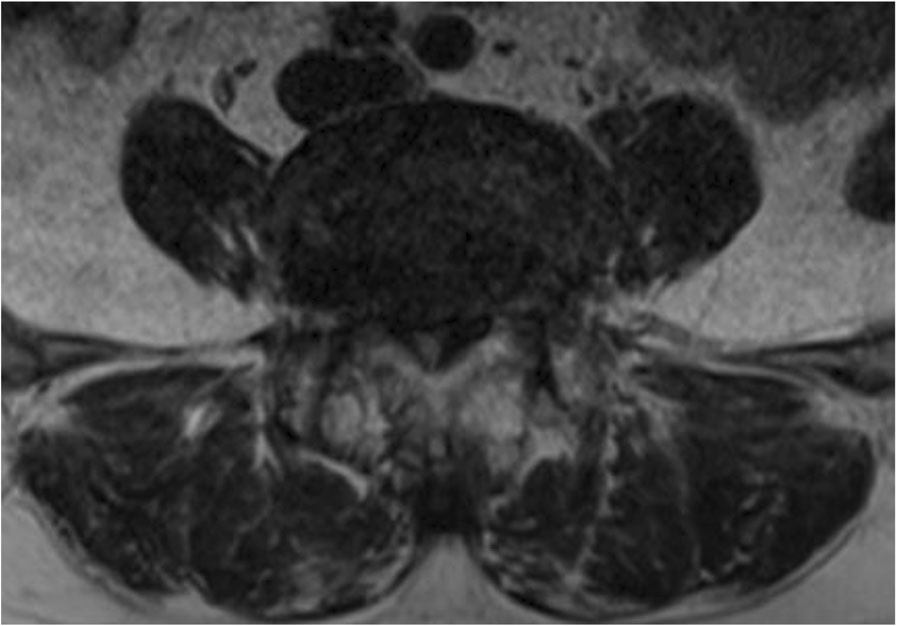
Extreme lumbar stenosis: Schizas stenosis grade D

LLIF, as an indirect decompression technique, does not directly remove a disc or osteophyte protruding into the spinal canal, and its decompression effect is not as thorough as traditional posterior decompression surgery. Radiographic studies have shown that improvement of the cross-sectional area of the spinal canal is significantly smaller after LLIF than after minimally-invasive transforaminal lumbar interbody fusion [3, 4]. Generally, extreme central canal stenosis, defined by a complete loss of cerebrospinal fluid signal on preoperative magnetic resonance imaging (MRI), was thought to be a relative contraindication for LLIF. According to Schizas’ classification [5], Grade D stenosis is defined as extreme stenosis, in which, in addition to no rootlets being recognizable, there is no epidural fat posterior to the dural sac (Fig. 1). Since patients with extreme stenosis (grade D) were excluded in most studies, the clinical and radiographic outcomes of LLIF for extreme lumbar spinal stenosis remain unknown. However, extreme lumbar spinal stenosis is common in clinical practice, especially in patients with multi-level degenerative lumbar disease. For the sake of reducing invasiveness, it is reasonable to perform LLIF for those patients instead of traditional open surgery, although additional posterior decompression is sometimes needed. The purpose of the current study was to evaluate the indirect neural decompression effect in patients with extreme lumbar spinal stenosis. In the current study, we compared the radiographic outcomes of LLIF for stenosis of Schizas grades A, B, C and D. Then, clinical outcomes of LLIF for a series of cases with stenosis of Schizas grade D were retrospectively evaluated.

## Methods

### Patients

Retrospectively, patients with a main diagnosis of degenerative lumbar spinal stenosis who underwent crenel lateral interbody fusion (CLIF) [6, 7], a modified extreme lateral interbody fusion technique, performed by our surgical group between June 2017 and December 2018 were reviewed. Patients who suffered from significant lumbar scoliosis, grade 2 spondylolisthesis, lumbar fracture or who had undergone prior lumbar surgery were excluded from this study. All the segments were grouped according to Schizas’ lumbar stenosis classification [5]. Grade A stenosis is the mildest, with abundant cerebrospinal fluid inside the dural sac. In grade B stenosis, the rootlets occupy the whole of the dural sac, but they can still be individualized. In grade C, no rootlets can be recognized but epidural fat can be visualized posteriorly. In grade D, in addition to no rootlets being recognizable, there is no epidural fat posteriorly.

### Radiological and clinical assessments

**Fig. 2:**
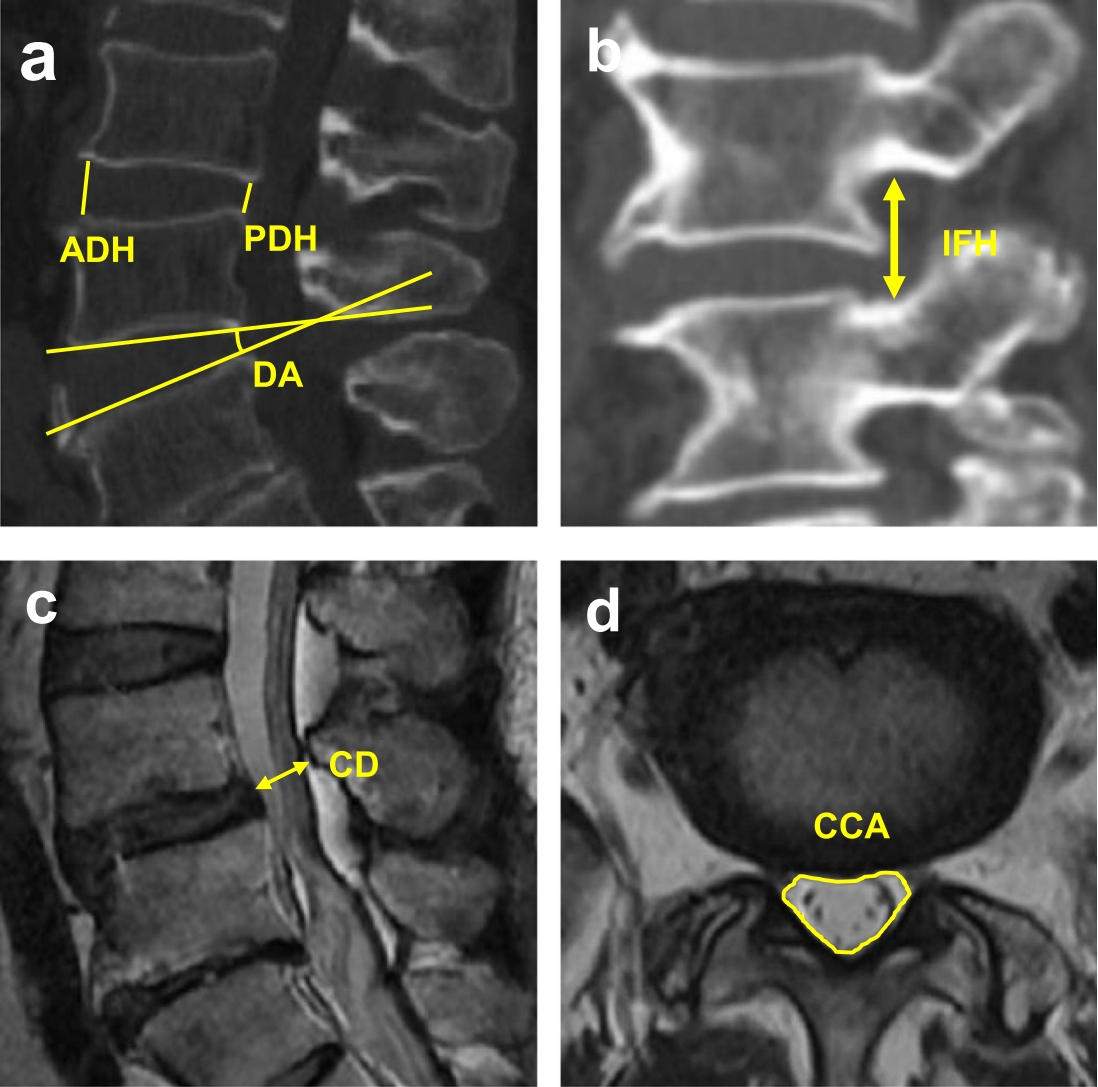
Measurement of radiographic parameters. ADH: anterior disc height. PDH: posterior disc height. DA: disc angle. IFH: intervertebral foramen height. CD: midsagittal canal diameter. CCA: axial central canal area

Standing lateral plain radiographs, MRI, and CT scans were obtained for all patients preoperatively and postoperatively. We measured the imaging data before and after the stage I CLIF (before the stage II posterior internal fixation). All radiographic parameters were measured using measurement tools on a medical center picture archiving and collecting system. The main measurement index included: the disc angle (DA), the anterior and posterior disk height (ADH and PDH), the bilateral intervertebral foramen height (IFH) on CT, and the midsagittal canal diameter (CD) and axial central canal area (CCA) on MRI (Fig. 2).

A total of 18 patients with at least one level with grade D stenosis who were followed for at least 6 months were clinically reviewed. The patients comprised eleven males and seven females, with a follow-up time of 19.61 ± 6.32 months (range: 9–26 months) (Table 1). Clinical outcomes were assessed by an experienced clinical research coordinator using a visual analogue scale (VAS) for back and leg pain as well as Oswestry Disability Index (ODI). The minimal clinically important difference for the ODI was 10 points [8]. These data were compared between before surgery and at the last follow-up. In addition, perioperative data and complications were recorded.

**Table 1:** Demographic characteristics of patients with Schizas’s classification D

Category	Values
Average age	67.94 ± 4.21
Gender(M:F)	11:7
BMI in kg/m2 (mean)	25.27 ± 2.45
Average surgical levels	1.78 ± 0.81
Disc with Schizas grade D	
L3/4	2
L4/5	16
VAS score for back	6.06 ± 1.35
VAS score for leg	5.39 ± 1.24
ODI score (%)	43.33 ± 7.32
Follow-up time (month)	19.61 ± 6.32

### Surgical techniques

The CLIF technique is a modified technique of extreme lateral lumbar interbody fusion (XLIF), aimed to minimize the approach-related complications of the traditional transpsoas approach [6]. This approach has some unique features which distinguish it from traditional XLIF. The psoas muscle working window was selected according to a safe working zone on axial MRI of the target intervertebral space, with the sagittal central line of the working zone located at least 1 cm anterior to the nerve root. The psoas muscle was split longitudinally along the muscle fiber until the lateral intervertebral space was visualized. In some cases, the genitofemoral nerve inside the psoas muscle was found and, if so, it was gently moved to the posterior with a small amount of muscle fiber. A novel designed retractor was positioned in the longitudinal direction to maintain the working window of the psoas muscle. Two vertebral screws were used to fix the retractors to the vertebral body as close as possible to the endplate, and then assemble the retractors to the fixed ring. The intervertebral space preparation and implant placement were consistent with the traditional LLIF.

During the second stage, usually 1 week after the first stage, additional direct posterior decompression was performed due to inadequate resolution of stenotic symptoms or radicular leg pain, and a positive straight leg raise test or femoral nerve stretch test. If direct decompression was required, open pedicle screws were applied, otherwise bilateral percutaneous screws were used.

### Statistics

Descriptive data are represented as means ± standard deviation (SD). Continuous variables were analyzed by 2-sample t test and paired t test. The data collected were processed using PASW Statistics 18.0. Values of *P* < 0.05 were considered to indicate statistical significance.

## Results

### Comparison of radiographic outcomes with other grades

Among the 181 segments included in this study, there were 23 (12.71%) segments of grade A, 37 (20.44%) segments of grade B, 103 (56.91%) segments of grade C and 18 (9.94%) segments of grade D. Overall, both the average ADH and PDH were significantly increased (Table 2). The average change of ADH was insignificantly greater than that of the PDH. Since the average preoperative PDH was significantly smaller than the ADH, the average change rate of PDH (56.29 ± 63.17%) was significantly larger than that of the ADH (31.53 ± 38.18%) (*P* < 0.001). The average increase of DA was 1.06 ± 3.84° (*P* = 0.005), which is small, but can partly be attributed to the greater improvement rate of PDH than ADH. Both the average left and right IFH were significantly increased (*P* < 0.001). The average change and change rate of the right IFH were not significantly greater than the left. In contrast, the average midsagittal CD and axial CCA on MRI were significantly increased (*P* < 0.001). The average change rate of midsagittal CD was 38.46 ± 66.27%. The average change rate of axial CCA was 28.03 ± 26.63%.

**Table 2:** Overall radiographic evaluation results

Results(*n* = 181)	preoperative	postoperative	Change	Change rate %	*P*
CT measurements					
Anterior disk height	10.96 ± 3.23	13.52 ± 2.54	2.57 ± 2.10	31.53 ± 38.18	<0.001
Posterior disk height	5.54 ± 2.21	7.74 ± 2.31	2.20 ± 1.53	56.29 ± 63.17	<0.001
Disc angle	5.96 ± 3.37	7.01 ± 3.69	1.06 ± 3.84	–	0.005
Left foraminal height	17.74 ± 2.35	19.63 ± 2.56	1.90 ± 1.76	11.22 ± 10.55	<0.001
Right foraminal height	17.75 ± 2.33	19.89 ± 2.53	2.14 ± 2.02	12.89 ± 13.07	<0.001
MRI measurements					
Midsagittal canal diameter	7.92 ± 2.86	10.02 ± 2.77	2.11 ± 1.63	38.46 ± 66.27	<0.001
axial central canal area	96.67 ± 50.12	117.60 ± 52.92	21.25 ± 18.30	28.03 ± 26.63	<0.001

This *P* value is the result of comparison between before and after surgery

**Fig. 3:**
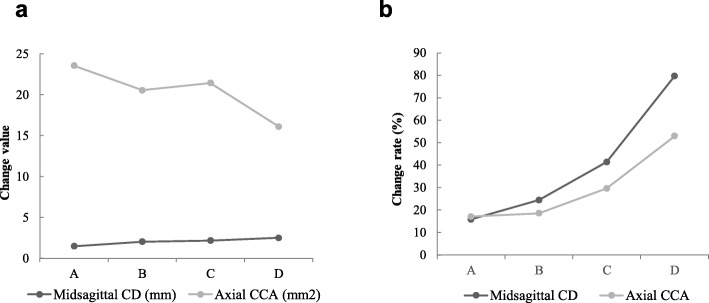
Changes of the spinal canal on MRI according to Schizas’ classification. The average change in the midsagittal midsagittal canal diameter (CD) of grade D was significantly greater than that of grade A, but showed no significant difference compared to grades B or C. The average change in the axial central canal area (CCA) of grade D was not significantly different from that in the other grades. However, the average change rate of midsagittal CD and axial CCA increased from grade A to grade D

With regard to the average change of midsagittal CD, the change in grade D was significantly greater than that in grade A, but did not differ significantly from grades B or C (Table 3). Interestingly, the average change rate of midsagittal CD increased from grade A to D, peaking at 79.69 ± 86.23% (Table 3 and Fig. 3). As to the average change of axial CCA, grade D did not differ significantly from the others. Likewise, the average change rate of axial CCA increased from grade A to D, peaking at 52.91 ± 34.41% (Table 3 and Fig. 3). The With regard to the average change of ADH, PDH, DA and IFH on both sides, grade D showed no significant difference compared with the others (Table 4).

**Table 3:** Summary of MRI evaluation

Parameter	Grade	preoperative	postoperative	Change value	Change rate %	*P*
Midsagittal canal diameter	A	10.96 ± 2.69	12.43 ± 2.45	1.48 ± 1.16	15.85 ± 17.58	0.011
	B	9.27 ± 2.35	11.30 ± 2.49	2.03 ± 1.69	24.42 ± 21.49	0.249
	C	7.37 ± 2.19	9.58 ± 2.29	2.18 ± 1.74	41.37 ± 76.16	0.362
	D	4.39 ± 2.52	6.89 ± 2.40	2.50 ± 1.25	79.69 ± 86.23	–
Axial central canal area	A	161.43 ± 56.25	185.00 ± 51.93	23.57 ± 15.14	17.12 ± 13.24	0.073
	B	128.11 ± 47.55	148.68 ± 49.18	20.57 ± 15.68	18.58 ± 15.51	0.222
	C	81.96 ± 25.90	103.40 ± 31.74	21.44 ± 20.07	29.64 ± 27.73	0.104
	D	32.61 ± 11.10	49.72 ± 17.47	16.11 ± 10.70	52.91 ± 34.41	–

This *P* value is the result of comparison of change value with grade D

**Table 4:** Summary of CT evaluation

Parameter	Grade	preoperative	postoperative	Change value	Change rate %	*P*
Anterior disk height	A	10.52 ± 3.06	13.39 ± 2.66	2.87 ± 2.24	33.27 ± 30.27	0.431
	B	10.51 ± 3.24	13.35 ± 2.35	2.84 ± 2.15	37.47 ± 45.17	0.407
	C	10.80 ± 3.31	13.26 ± 2.59	2.47 ± 2.07	31.57 ± 39.95	0.803
	D	12.89 ± 2.87	15.22 ± 2.34	2.33 ± 2.06	21.39 ± 20.73	–
Posterior disk height	A	4.91 ± 1.90	7.57 ± 2.02	2.65 ± 1.64	71.46 ± 69.84	0.979
	B	5.62 ± 2.53	8.32 ± 2.40	2.70 ± 1.47	69.14 ± 71.19	0.940
	C	5.56 ± 2.22	7.40 ± 2.33	1.83 ± 1.43	47.03 ± 57.90	0.070
	D	6.17 ± 1.91	8.83 ± 1.95	2.67 ± 1.75	55.97 ± 57.35	–
Disc angle	A	5.50 ± 3.34	5.71 ± 2.61	1.21 ± 3.68	–	0.852
	B	5.04 ± 3.41	5.90 ± 3.78	0.85 ± 3.97	–	0.906
	C	6.09 ± 3.32	7.34 ± 3.80	1.26 ± 3.91	–	0.790
	D	6.74 ± 3.63	7.73 ± 3.89	0.99 ± 3.89	–	–
Left foraminal height	A	17.43 ± 2.27	19.74 ± 2.49	2.30 ± 2.12	14.13 ± 13.97	0.751
	B	17.86 ± 2.49	20.36 ± 2.96	2.50 ± 1.62	14.25 ± 9.23	0.433
	C	17.71 ± 2.43	19.24 ± 2.53	1.53 ± 1.67	9.19 ± 9.93	0.206
	D	18.17 ± 1.82	20.28 ± 1.60	2.11 ± 1.75	12.20 ± 10.28	–
Right foraminal height	A	16.65 ± 2.27	19.70 ± 2.20	3.04 ± 1.87	19.28 ± 12.94	0.098
	B	17.68 ± 2.14	20.51 ± 2.66	2.84 ± 1.83	16.68 ± 13.08	0.146
	C	17.96 ± 2.36	19.62 ± 2.58	1.66 ± 2.05	9.95 ± 12.78	0.414
	D	18.39 ± 2.09	20.44 ± 2.15	2.05 ± 1.83	11.83 ± 10.82	–

This *P* value is the result of comparison of change value with grade D

### Clinical outcome of patients with extreme degenerative lumbar stenosis

**Fig. 4:**
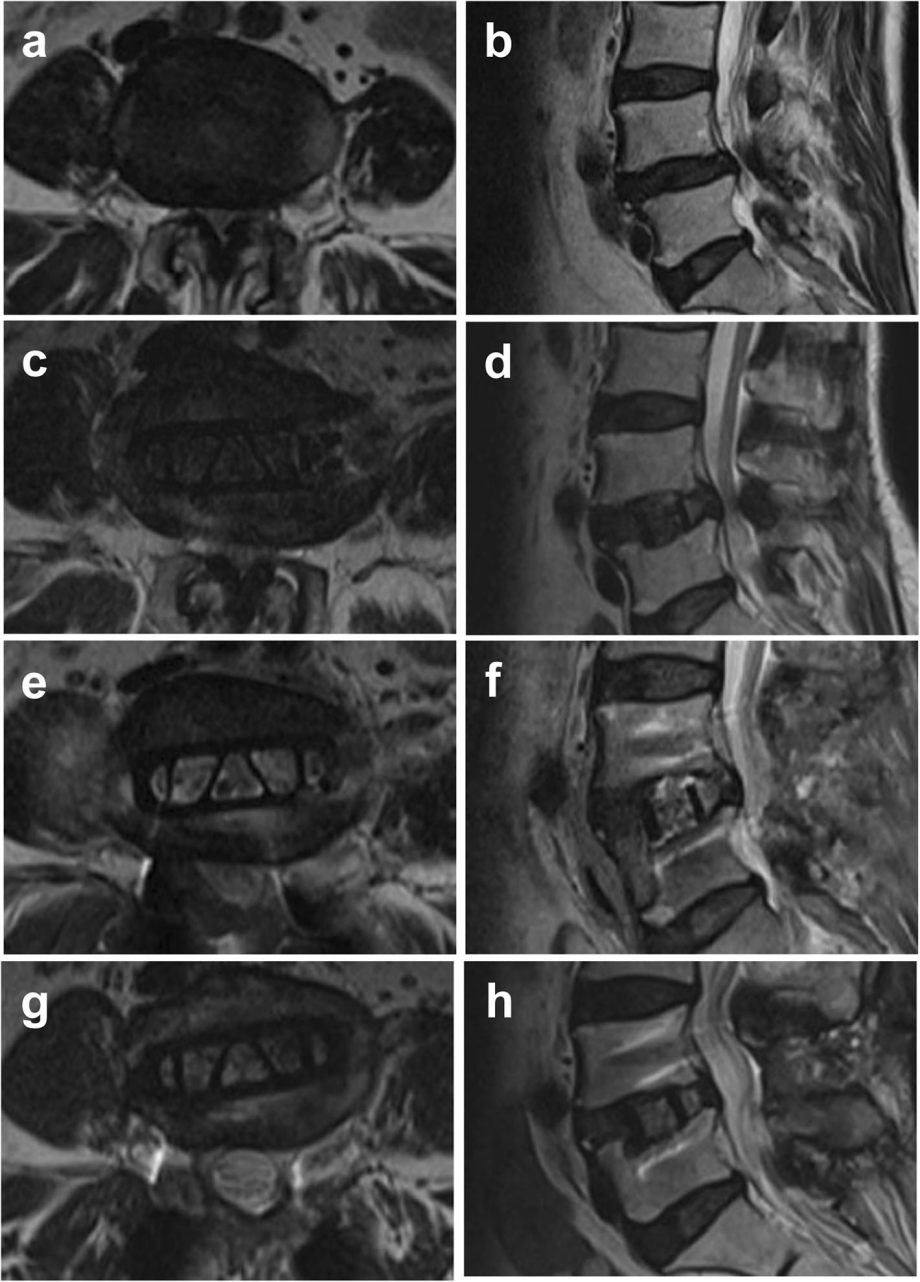
A 66-year-old woman with Schizas grade D preoperatively at L4/5 (**a**, **b**). Her axial central canal area and midsagittal canal diameter partially improved after CLIF surgery (**c**, **d**) and significantly improved after second-stage laminectomy (**e**, **f**). Neurological decompression was maintained 15 months after surgery (**g**, **h**)

**Fig. 5:**
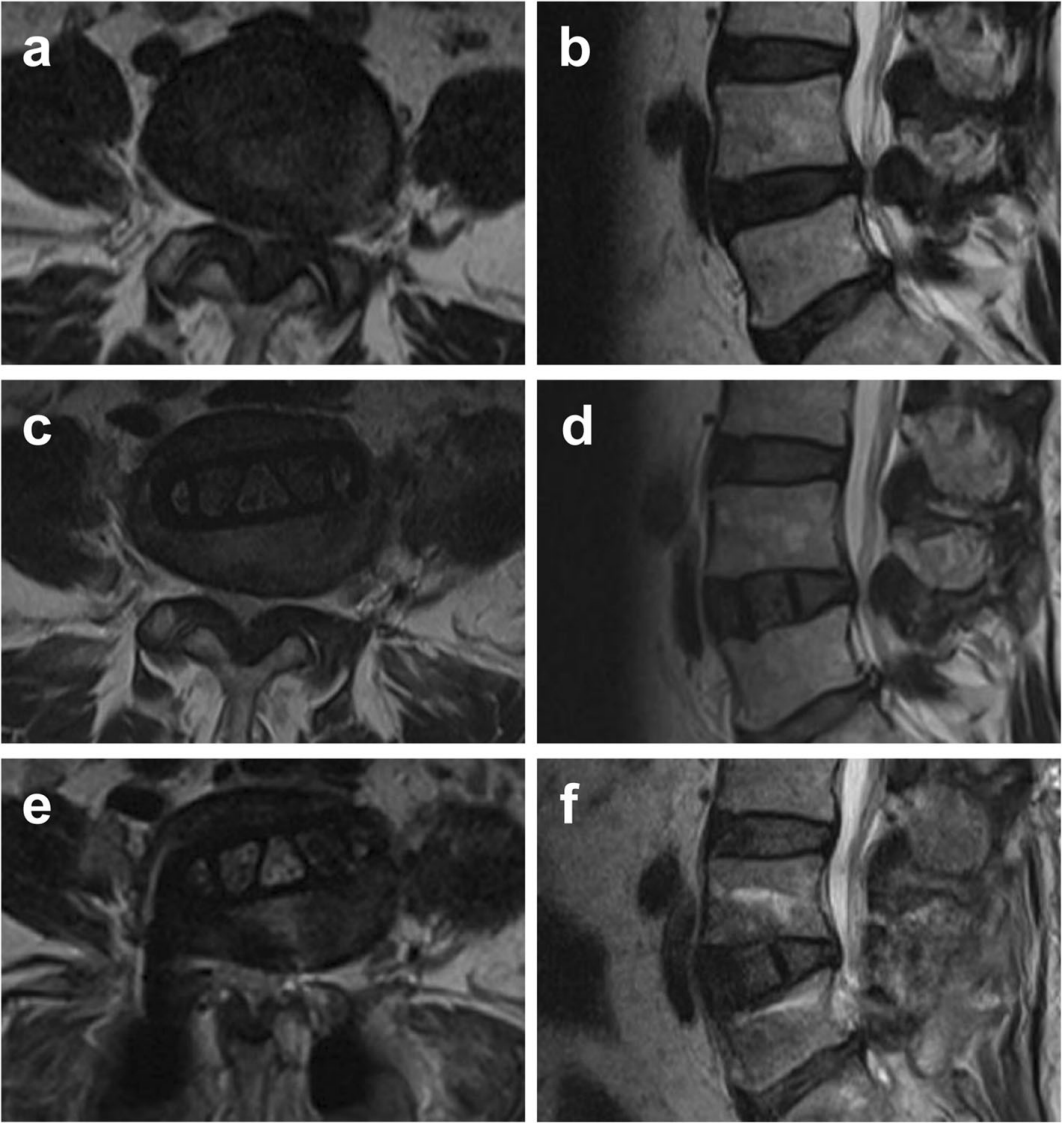
A 70-year-old woman with Schizas grade D and severe ligamentum flavum hypertrophy preoperatively at L4/5 (**a**, **b**). Her axial central canal area and midsagittal canal diameter achieved small improvements after CLIF surgery with the presence of ligamentum flavum hypertrophy (**c**, **d**). Significant improvement was achieved after second-stage laminectomy (**e**, **f**)

Eighteen patients with at least one level of grade D who underwent CLIF were clinically reviewed. All of them were retrospectively followed-up, with a mean follow-up time of 19.61 ± 6.32 months. The clinical follow-up analysis revealed a statistically-significant improvement of established outcome scores. The mean ODI improved from 43.33 ± 7.32% preoperatively to 22.56 ± 8.63% at the last follow-up (*P* < 0.001). In a similar manner, the VAS for back decreased from 6.06 ± 1.35 to 2.39 ± 0.78 (*P* < 0.001), while the VAS for leg decreased from 5.39 ± 1.24 to 1.89 ± 1.02 (*P* < 0.001). Sixteen of 18 segments (88.89%) with grade D underwent posterior decompression (Figs. 4 and 5). One patient who had received a stand-alone CLIF surgery had cage subsidence and presented with worsening back pain and neurological function at 2 months after surgery. However, she refused to undergo a posterior decompression. At the last follow-up, although she complained about back pain, her VAS scores of both back and leg pain had decreased from 6 preoperatively to 4. Her ODI score was slightly decreased from 51.11% preoperatively to 40%. Still, she refused to undergo a posterior decompression procedure.

**Fig. 6:**
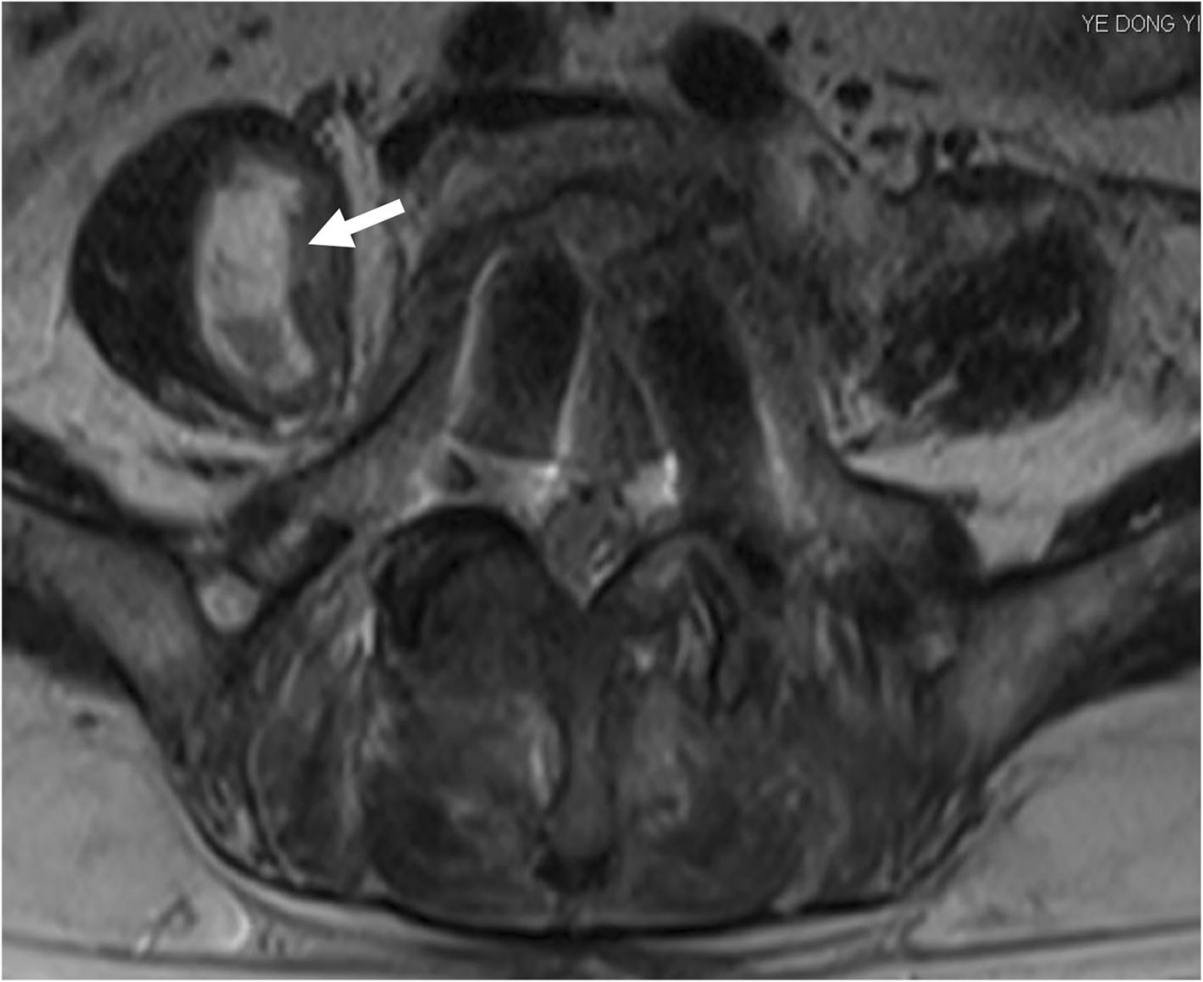
A 66-year-old woman suffering from contralateral iliopsoas hematoma (arrow)

In this group, seven patients (38.89%) presented with surgery-related complications. A total of 11 complications occurred in seven patients. Pain in the front of the thigh was reported in five cases, and numbness was observed in three cases. Muscle weakness of the psoas major muscle was decreased in two cases. One patient suffered from deep venous thrombosis and interventional therapy was performed. There were no complications such as knee extension weakness, vascular injury, sympathetic nerve injury, visceral injury or ureteral injury in this series.

In the five patients with anterior thigh pain, the mean VAS score for the leg was 3.20 + 0.84 (range: 2–4 points) immediately postoperatively, but the pain had subsided 2–3 months later. In the two patients with hip flexion weakness, the strength of the psoas muscle was grade 3 and 4 immediately postoperatively, but recovered to grade 4 and 5 respectively 3 months later. One of these patients suffered from psoas hematoma which was relieved after conservative treatment (Fig. 6).

## Discussion

The current study shows that the radiographic decompression effect of LLIF for Schizas grade D segments was comparable to the effect on other grades. However, patients with extreme lumbar spinal stenosis are not good candidates for LLIF alone. Stand-alone LLIF is not suggested for such patients, but with concomitant posterior decompression, LLIF can achieve a satisfactory clinical outcome for extreme lumbar spinal stenosis.

Although the indirect neural decompression effect of LLIF for lumbar stenosis has been addressed in previous studies [3, 4, 9–13], the purpose of the current study was to evaluate the indirect neural decompression effect in patients with extreme lumbar spinal stenosis. The average changes in CD and CCA of grade D were 2.50 ± 1.25 mm and 16.11 ± 10.70 mm^2^, which were comparable with the effects in other grades, and indicated that the indirect decompression effect is not compromised in patients with extreme spinal stenosis. However, the average rates of change CD and CCA for grade D were 79.69 ± 86.23% and 52.91 ± 34.41% respectively, both of which increased from grade A to D (Fig. 3. B), and in accordance with Fujibayashi’s finding [14] that the greater the stenosis preoperatively, the greater the improvement rate in neural decompression with LLIF compared with milder stenosis. Oliviera et al. [11] described increases of 2.4 mm (33.1%) and 12.4 mm^2^ (8.4%), respectively, in CD and CCA after XLIF. Elowitz et al. [15] found a 3.8 mm improvement in anterior–posterior diameter of the dural sac, and the area of the dural sac increased an average of 83 mm^2^ (143%) after XLIF. Castellvi et al. [12] found that the CCA was improved by 10 mm^2^ (27%) and 23 mm^2^ (17%) at 3 months and 1 year, respectively, after XLIF. At the 3-month follow-up, Isaacs et al. [3] found an increase in the CCA of 20.8 mm^2^ and in the CD of 1.2 mm after XLIF. With the exception of the results reported by Elowitz et al. [15], the improvement of central canal stenosis in patients with Schizas grade D observed in our study is comparable with those studies [3, 11, 12].

With regard to the indirect decompression effect on foraminal stenosis, Oliviera et al. [11] described an increase of 2.48 mm (13.1%) of foraminal height after XLIF. In another retrospective study with 90 patients undergoing LLIF, Alimi et al. [16] found foraminal height increased by 3.1 mm (20%). At 3-month follow-up, Isaacs et al. [3] found an increase in the approach-side foraminal height of 2.16 mm and in the contralateral-side foraminal height of 1.39 mm after XLIF. For segments with Schizas grade D, we found an increase of foraminal height of 2.11 ± 1.75 mm (12.20 ± 10.28%) on the left side (approach-side) and 2.05 ± 1.83 mm (11.83 ± 10.82%) on right side, which is comparable with those studies and not significantly different from the improvement seen with other grades. Likewise, regarding the change of anterior and posterior disk height and segment angle, grade D showed no significant difference from other grades. Thus, we consider that preoperative central canal stenosis does not significantly influence the degree of change of indirect decompression after LLIF.

In our group, the mean ODI and VAS scores were both significantly improved at the last follow-up. Post-operatively, the average axial CCA of grade D was 49.87 ± 18.81 mm^2^, which was significantly smaller than the average preoperative axial CCA of grade C (82.06 ± 26.97 mm^2^). Sixteen of 18 (88.89%) segments with stenosis of grade D received posterior direct decompression. We believed that additional posterior decompression after LLIF was important to ensure sufficient decompression in patients with extreme lumbar spinal stenosis. In addition, it is extremely dangerous to perform posterior instrumentation without direct decompression in patients with severe stenosis exhibiting preoperative paralysis [17]. The clinical indications for posterior decompression after LLIF were inconsistent. There have been studies which claimed that factors likely to cause failure of indirect decompression include cage subsidence, low bone mineral density, severe central canal stenosis, ligamentum flavum hypertrophy, and osteophytes in the lateral recess and foraminal canal [11, 14, 18–22]. Among them, severe central canal stenosis might be the major risk factor. Nakashima et al. [17] claimed that patients with preoperative lower limb paralysis and severe stenosis were at a higher risk of perioperative neurological deterioration and that this was particularly true for patients exhibiting ligament ossification around the spinal canal. Moreover, factors that are less likely to influence indirect decompression in LLIF are cage position, cage type, side of approach, preoperative sagittal/coronal alignment, presence of facet arthropathy, spinal level (upper or lower lumbar spine), and number of operated spinal levels [9, 21, 23–26].

In the current group, seven out of eight patients with extreme lumbar stenosis who underwent single-level CLIF received second-stage posterior decompression. The patient who had received a stand-alone CLIF surgery had cage subsidence and presented with worsening back pain and neurological function at 2 months after surgery. Lack of posterior supplemental fixation may lead to a loss of acquired indirect decompression after the operation. Thus, we do not suggest stand-alone surgery for patients with extreme spinal stenosis. Posterior lumbar interbody fusion may be a better surgical option for patients with single-level extreme lumbar spinal stenosis.

There are some limitations to this study, including the retrospective nature of the study, the limited follow-up, and the small sample size of grade D. Since we did not collect all their radiographic data during follow-up, we could not show the radiographic changes. The clinical outcomes of patients with extreme lumbar stenosis were not compared with those with mild lumbar stenosis.

## Conclusions

The radiographic decompression effect of LLIF for Schizas grade D segments was comparable with that of other grades. Patients with extreme lumbar spinal stenosis are not good candidates for LLIF alone. Posterior decompression was necessary for LLIF to achieve a satisfactory clinical outcome for extreme lumbar spinal stenosis of Schizas grade D.

## Data Availability

The datasets used and/or analyzed during the current study are available from the corresponding author on reasonable request.

## Abbreviations

LLIF: Lateral Lumbar Interbody Fusion
CLIF: Crenel Lateral Interbody Fusion
MRI: Magnetic Resonance Imaging
ADH: Anterior Disk Height
PDH: Posterior Disk Height
DA: Disc Angle
IFH: Intervertebral Foramen Height
CD: Canal Diameter
CCA: Central Canal Area
ODI: Oswestry Disability Index
VAS: Visual Analogue Scale
